# Mechanisms of splicing-dependent *trans*-synaptic adhesion by PTPδ–IL1RAPL1/IL-1RAcP for synaptic differentiation

**DOI:** 10.1038/ncomms7926

**Published:** 2015-04-24

**Authors:** Atsushi Yamagata, Tomoyuki Yoshida, Yusuke Sato, Sakurako Goto-Ito, Takeshi Uemura, Asami Maeda, Tomoko Shiroshima, Shiho Iwasawa-Okamoto, Hisashi Mori, Masayoshi Mishina, Shuya Fukai

**Affiliations:** 1 Structural Biology Laboratory, Life Science Division, Synchrotron Radiation Research Organization and Institute of Molecular and Cellular Biosciences, The University of Tokyo, Tokyo 113-0032, Japan; 2 Department of Medical Genome Sciences, Graduate School of Frontier Sciences, The University of Tokyo, Chiba 277-8501, Japan; 3 CREST, JST, Saitama 332-0012, Japan; 4 Department of Molecular Neuroscience, Graduate School of Medicine and Pharmaceutical Sciences, University of Toyama, Toyama 930-0194, Japan; 5 Department of Molecular Neurobiology and Pharmacology, Graduate School of Medicine, The University of Tokyo, Tokyo 113-0033, Japan; 6 PRESTO, JST, Saitama 332-0012, Japan; 7 Department of Molecular and Cellular Physiology, Shinshu University School of Medicine, Nagano 390-8621, Japan; 8 Institute for Biomedical Sciences, Interdisciplinary Cluster for Cutting Edge Research, Shinshu University, Nagano 390-8621, Japan; 9 Brain Science Laboratory, The Research Organization of Science and Technology, Ritsumeikan University, Shiga 525-8577, Japan

## Abstract

Synapse formation is triggered through *trans*-synaptic interaction between pairs of pre- and postsynaptic adhesion molecules, the specificity of which depends on splice inserts known as ‘splice-insert signaling codes'. Receptor protein tyrosine phosphatase δ (PTPδ) can bidirectionally induce pre- and postsynaptic differentiation of neurons by *trans*-synaptically binding to interleukin-1 receptor accessory protein (IL-1RAcP) and IL-1RAcP-like-1 (IL1RAPL1) in a splicing-dependent manner. Here, we report crystal structures of PTPδ in complex with IL1RAPL1 and IL-1RAcP. The first immunoglobulin-like (Ig) domain of IL1RAPL1 directly recognizes the first splice insert, which is critical for binding to IL1RAPL1. The second splice insert functions as an adjustable linker that positions the Ig2 and Ig3 domains of PTPδ for simultaneously interacting with the Ig1 domain of IL1RAPL1 or IL-1RAcP. We further identified the IL1RAPL1-specific interaction, which appears coupled to the first-splice-insert-mediated interaction. Our results thus reveal the decoding mechanism of splice-insert signaling codes for synaptic differentiation induced by *trans*-synaptic adhesion between PTPδ and IL1RAPL1/IL-1RAcP.

## Abstract (web summary)

Synapse formation depends on *trans*-synaptic interactions between selective pairs of pre- and postsynaptic adhesion molecules. Here, the authors establish the basis for the splice isoform-dependent selectivity of receptor protein tyrosine phosphatase δ as it forms *trans*-synaptic contacts with IL1RAPL1 and IL-1RAcP.

## Graphical abstract

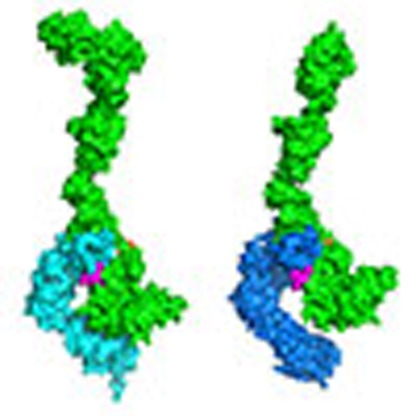

Mammalian brains are composed of at least a few hundred billions of neurons, which are connected by synapses in a spatiotemporally organized manner to establish higher-order brain functions such as learning, memory and emotion. Synapse formation is triggered through *trans*-synaptic interactions between selective pairs of pre- and postsynaptic adhesion molecules known as ‘synaptic organizers' or ‘synaptogenic proteins', many of which are associated with neurodevelopmental disorders such as intellectual disability and autism^1^,^2^,^3^,^4^,^5^,^6^. Recent studies have highlighted a major role of type-IIa receptor protein tyrosine phosphatases (RPTPs) as synaptic organizers in pre- and postsynaptic differentiation during neural development^5^,^6^. Vertebrate type-IIa RPTPs comprise LAR, PTPσ and PTPδ, which share common domain architecture with a single transmembrane (TM) helix flanked by a large extracellular domain (ECD) and cytoplasmic tandem PTP domains: the membrane-proximal PTP domain (D1) is catalytically active, whereas the membrane-distal PTP domain (D2) is inactive. The ECD consists of three immunogloblin-like (Ig) domains and four to eight fibronectin type-III (Fn) domains. In mammals, presynaptic type-IIa RPTPs form *trans*-synaptic adhesion by specifically binding to their cognate postsynaptic organizers through their ECDs for inducing synaptic differentiation of neurons. To date, five different types of postsynaptic organizers for type-IIa RPTPs have been reported: interleukin-1 receptor (IL-1R) accessory protein (IL-1RAcP)^7^, IL-1RAcP-like-1 (IL1RAPL1)^8^, neurotrophin receptor tyrosine kinase C (TrkC)^9^, netrin-G ligand-3 (NGL-3)^10^,^11^ and Slit- and Trk-like (Slitrk) family proteins^12^,^13^. For example, co-culture with either IL-1RAcP- or IL1RAPL1-expressing non-neuronal HEK293T cells can stimulate presynaptic differentiation in contacting axons of cultured cortical neurons, however, the IL1RAPL1- and IL-1RAcP-induced presynaptic differentiations are completely abolished and decreased by ∼70% in the neurons lacking PTPδ, respectively^7^,^8^. Similarly, PTPδ can induce postsynaptic differentiation of cultured cortical neurons in a manner specific to IL1RAPL1 and IL-1RAcP (more precisely, a central nervous system-restricted IL-1RAcP isoform, which differs only in the carboxy (C)-terminal from a widely distributed isoform). On the other hand, Slitrk family proteins induce presynaptic differentiation through the interaction with type-IIa RPTPs^12^,^13^. Among Slitrk family proteins, Slitrk3 selectively induces inhibitory synapse formation, suggesting potential roles of synapse organizers in balancing excitatory and inhibitory synapses^12^. Various combinations of *trans*-synaptic adhesion between pre- and postsynaptic organizers might play an important role in generating extremely diverged but highly organized synaptic connections of neurons.

Varieties of the combinations between pre- and postsynaptic organizers rely upon structural diversities of their ECDs, which can be drastically increased by multiple isoforms and splice variants. In fact, several isoforms and splice variants of type-IIa RPTPs are expressed in mouse brain, and bind selectively to the corresponding postsynaptic organizers^5^,^6^. Type-IIa RPTPs commonly have three Ig domains (Ig1–Ig3) in their amino (N)-terminal regions. The first splicing site is located within Ig2, whereas the second one is in the junction between Ig2 and Ig3. These two splicing sites generate variants of the type-IIa RPTPs that lack or contain short-peptide inserts termed mini-exon peptides. A recent study has shown that Slitrk family members bind to all type-IIa RPTP members, depending on the second mini-exon peptide (meB)^14^. On the other hand, IL1RAPL1 and IL-1RAcP specifically bind to PTPδ but not to LAR or PTPσ. Furthermore, our previous cell-surface binding assays showed that both the first mini-exon peptide (meA) and meB can alter their binding properties^7^,^8^. MeB comprises four residues, Glu-Leu-Arg-Glu, whereas there are three variations in meA: a three-residue peptide Glu-Ser-Ile (meA3), a six-residue peptide Gly-Gly-Thr-Pro-Ile-Arg (meA6) and their tandem combination (Glu-Ser-Ile-Gly-Gly-Thr-Pro-Ile-Arg) (meA9). Binding to IL-1RAcP is significantly enhanced by the presence of both meA and meB, independently of the meA variation. In contrast, the length and sequence of meA are critically important for binding to IL1RAPL1. Only PTPδ variants containing meA9 and meA6 can bind to IL1RAPL1. The meB insertion increases binding of the meA9-containing variant, whereas the meA6-containing variant absolutely requires the combination with meB. These findings indicate that information of synaptogenic postsynaptic ligands for the type-IIa RPTPs is encoded in their splice inserts. Similarly, in another presynaptic organizer neurexin, the insert in the splice site 4 regulates the specificity to postsynaptic organizers Cbln1-GluRδ2 and LRRTM1/2 (refs 15, 16). The ‘splice-insert signaling code' concept for specific synaptic connections was proposed originally for *trans*-synaptic adhesion between neurexin and neuroligin, but appears likely more general for synaptogenic adhesion^3^,^7^,^8^,^9^,^15^,^16^,^17^,^18^. However, structural mechanisms underlying this concept remain elusive. To elucidate the structural basis for decoding the ‘splice-insert signaling code' in the type-IIa RPTPs, we determined the crystal structures of PTPδ-ECD in complex with IL-1RAcP-ECD and IL1RAPL1-ECD. Together with structure-based mutational analyses by surface plasmon resonance (SPR) spectroscopy and synaptogenic assays, we reveal the structural basis of the splicing-dependent *trans*-synaptic adhesion by PTPδ and IL1RAPL1/IL-1RAcP for synaptic differentiation.

## Results

### Structure of the complex between PTPδ and IL1RAPL1

**Figure 1:**
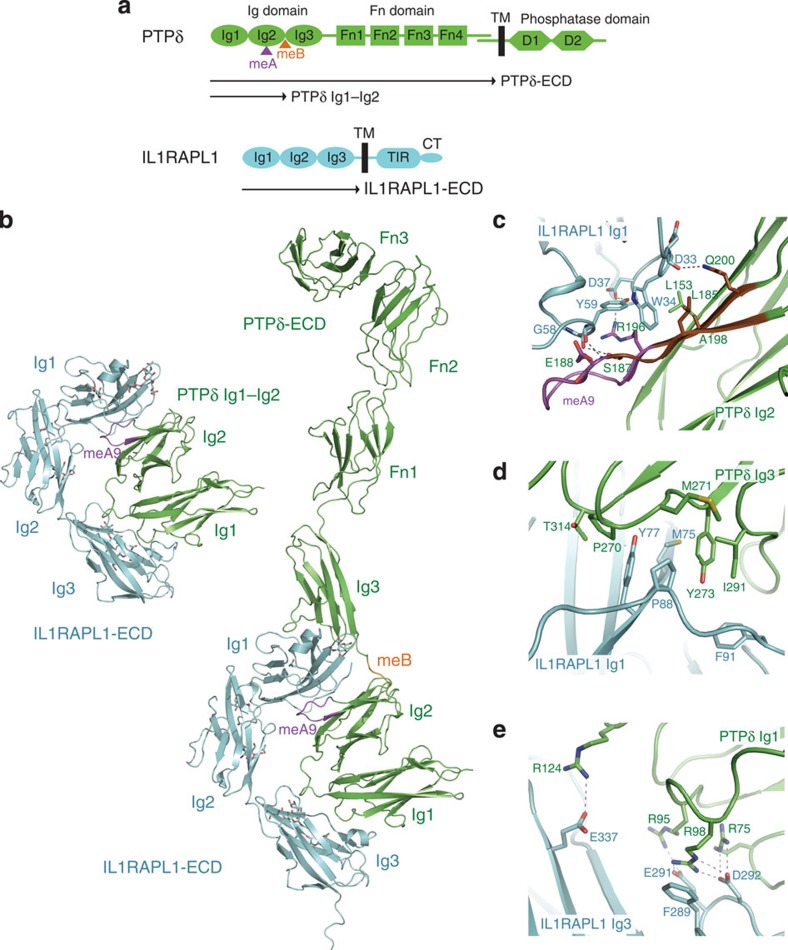
Structure of the complex between PTPδ and IL1RAPL1. (**a**) Domain organizations of PTPδ and IL1RAPL1. The meA and meB insertion sites of PTPδ are indicated by magenta and orange triangles, respectively. (**b**) Overall structures of the PTPδ Ig1–Ig2
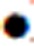
IL1RAPL1-ECD and PTPδ-ECD
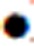
IL1RAPL1-ECD complexes. PTPδ and IL1RAPL1 are coloured green and cyan, respectively. The meA9 and meB insertions are highlighted in magenta and orange, respectively. *N*-linked glycans are shown as sticks. (**c**) Interactions between the Ig1 domain of IL1RAPL1 and the Ig2 domain of PTPδ. The colouring scheme is the same as that in **b**, except that IL-1RAcP/IL1RAPL1-Ig1-interacting β-strands of PTPδ are coloured brown. Residues involved in the interactions are shown as sticks. Hydrogen bonds are indicated by dotted lines. (**d**) Hydrophobic interactions between the Ig1 domain of IL1RAPL1 and the Ig3 domain of PTPδ. The representation scheme is the same as that in **c**. (**e**) Interactions between the Ig3 domain of IL1RAPL1 and the Ig1 domain of PTPδ. The representation scheme is the same as that in **c**.

To elucidate the structural basis of the PTPδ–IL1RAPL1 interaction for *trans*-synaptic adhesion, we determined the crystal structures of IL1RAPL1-ECD in complex with PTPδ Ig1-Ig2 and the full-length PTPδ-ECD at 2.7 and 4.4 Å resolutions, respectively (Fig. 1a,b, Table 1). Description of PTPδ Ig1-Ig2 will be based on the 2.7-Å-complex structure, whereas that of the other regions of PTPδ-ECD will be based on the 4.4-Å-complex structure. For crystallography, we used a PTPδ isoform containing four Fn domains and both meA9 and meB, which is the most abundant in the developing brain and has the highest affinity for IL1RAPL1 (*K*_d_: 0.15 μM) among all the PTPδ variants (Table 2). Hereafter, ‘PTPδ' indicates this isoform, unless otherwise noted. The Ig1-Ig2 domains of PTPδ retain the binding ability for IL1RAPL1-ECD, as shown in our serial deletion analysis of PTPδ (Supplementary Fig. 1a). PTPδ-ECD binds to IL1RAPL1-ECD at a ratio of 1:1 and the asymmetric unit contains one PTPδ
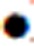
IL1RAPL1 complex (Fig. 1b and Supplementary Fig. 1b). IL1RAPL1-ECD consists of three Ig domains, which are arranged in an L-shape (Fig. 1a,b). PTPδ-ECD exhibits an elongated shape with a length of 180 Å along the longest axis. The Ig1 and Ig2 domains of PTPδ tightly interact with each other to form a compact V-shaped unit, which is almost identical to the isolated Ig1-Ig2 structures of PTPδ^19^ (PDB:2YD6, 2YD7) (rmsd values of 0.8–0.9 Å for 195 Cα atoms). No substantial change in the backbone structure of PTPδ Ig1-Ig2 occurred upon binding to IL1RAPL1 (Supplementary Fig. 1c). The Ig3 domain is spatially separated by the meB-containing linker and positioned apart from the Ig1-Ig2 unit. The following Fn1 and Fn2 domains are aligned in a straight line, similarly to the isolated PTPδ Fn1-Fn2 structure, which we determined at 2.0 Å resolution (Supplementary Fig. 1d). The Fn3 domain of PTPδ is oriented perpendicular to the Fn1-Fn2 domains, as observed in the PTPσ Ig1-Fn3 structure (Supplementary Fig. 1e). Electron density of the Fn4 domain and subsequent C-terminal region was not visible, likely due to the disorder. The length of the PTPδ-ECD·IL1RAPL1-ECD complex along the longest axis is 206 Å, which is equivalent to the average length of excitatory synaptic clefts, implying that PTPδ-ECD is kinked at the junction between the Fn2 and Fn3 domains in synapses.

### Interactions between IL1RAPL1 Ig1 and PTPδ Ig2 domains

The Ig1 domain of IL1RAPL1 interacts with both Ig2 and Ig3 domains of PTPδ (Fig. 1b). The interface between the IL1RAPL Ig1 and PTPδ Ig2 domains comprises both hydrophobic and hydrophilic interactions with a buried surface area of 699 Å^2^. These interactions occur on two β-strands comprising Arg181–Ser187 and Arg196–Glu202 in the Ig2 domain of PTPδ, between which meA9 is inserted (Fig. 1c). These two β-strands are hereafter referred to as the IL1RAPL1/IL-1RAcP-Ig1-interacting β-strands, because they also mediate the interaction with the Ig1 domain of IL-1RAcP as described later. The first eight residues of meA9 (^188^Glu-Ser-Ile-Gly-Gly-Thr-Pro-Ile^195^) form a loop connecting these two β-strands, whereas the last residue, Arg196, extends one of the two β-strands. Trp34 of IL1RAPL1 hydrophobically interacts with Leu153, Ala198 and Leu185 of PTPδ (Fig. 1c). The affinity of the IL1RAPL1 W34A mutant for PTPδ could not be measured by surface-plasmon resonance (SPR) spectroscopy, due to substantially low signals, indicating that this hydrophobic interaction is essential for binding between PTPδ and IL1RAPL1. The aliphatic portion of PTPδ Arg196 also participates in this hydrophobic interaction, which enables the Ig2 domain of PTPδ to more tightly interact with the Ig1 domain of IL1RAPL1 (Fig. 1c). The Arg196 side chain forms a hydrogen bond with Asp37 of IL1RAPL1, which is further stabilized by a hydrogen bond with Tyr59 of IL1RAPL1. Concomitantly, the side chain of Ser187 and the main-chain N atom of Glu188 in meA9 hydrogen bond with the main-chain O atoms of IL1RAPL1 Tyr59 and Gly58, respectively. The interactions mediated by Arg196 of PTPδ are critically important: the R196A mutation of PTPδ and the D37A mutation of IL1RAPL1 decreased the affinity 11- and 16-fold, respectively (Table 3). The meA6-containing PTPδ variant also contains Arg196 but has a fivefold reduced affinity for IL1RAPL1 (Table 2). Therefore, this affinity reduction of the meA6-containing variant might be due to a lack of the concomitant hydrogen bonding. The atomic-level meA-specific interactions described here illustrate how the meA insertion contributes to the affinity between PTPδ and IL1RAPL1.

### Relationship of meB with IL1RAPL1 Ig1–PTPδ Ig3 interaction

On the opposite side to the IL1RAPL1 Ig1–PTPδ Ig2 interface, the Ig1 domain of IL1RAPL1 interacts with the Ig3 domain of PTPδ with a buried surface area of 694 Å^2^. The IL1RAPL1 Ig1–PTPδ Ig3 interface is stabilized by hydrophobic interactions (Fig. 1d). The side chains of IL1RAPL1 Tyr77 and PTPδ Tyr273 are aligned in an antiparallel manner to form a hydrophobic core, which is surrounded by Met75, Pro88 and Phe91 of IL1RAPL1 and Pro270, Met271, Ile291 and Thr314 of PTPδ. The Y273A mutation of PTPδ decreased the affinity for IL1RAPL1 sixfold, whereas the M75A/Y77A/P88A/F91A quadruple mutation of IL1RAPL1 decreased the affinity for PTPδ 19-fold (Table 3).

The meB insertion of PTPδ is important for binding between PTPδ and IL1RAPL1. The lack of meB reduces the affinity fivefold (Table 2). In the present PTPδ-ECD
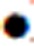
IL1RAPL1-ECD complex, the meB insertion appears to adjust the relative spacing and orientation between the Ig2 and Ig3 domains of PTPδ (Fig. 1b). When the apo structure of PTPδ Ig1-Fn2 containing meA3 and lacking meB (PTPδA3B^−^) (Supplementary Fig. 1f), which we determined at 3.5 Å resolution, is superposed onto the IL1RAPL1-ECD-bound structure of PTPδ Ig1-Fn2 (Supplementary Fig. 1g) using the Ig1–Ig2 unit of PTPδ as the reference, the Ig3 domain of the apo PTPδA3B^−^ is oriented in the opposite direction from that of the IL1RAPL1-ECD-bound PTPδ and cannot interact with IL1RAPL1, even if structural flexibility of the linker between the Ig2 and Ig3 domains of PTPδ is assumed (Supplementary Fig. 1g). Replacement of the native meB (Glu-Leu-Arg-Glu) by two tandem units of meB or a Gly-Ser-Ser-Gly or Gln-Leu-Glu-Gln tetrapeptide hardly affected the affinity, whereas that by three tandem units reduced the affinity threefold (Table 3). Insertions of four or more residues at the meB position are sufficient for binding to IL1RAPL1, but the longer insertion prevents the efficient binding. Therefore, the meB insertion likely acts as an adjustable linker to locate the Ig3 domain of PTPδ in the appropriate position for interacting with the Ig1 domain of IL1RAPL1, representing how the meB insertion contributes to the affinity between PTPδ and IL1RAPL1.

### Interactions between IL1RAPL1 Ig3 and PTPδ Ig1 domains

The Ig3 domain of IL1RAPL1 interacts with the Ig1 domain of PTPδ with a buried surface area of 433 Å^2^. The IL1RAPL1 Ig3–PTPδ Ig1 interface is overlapped with the putative binding pocket for glycosaminoglycan chains of heparan sulfate and chondroitin sulfate proteoglycans (Supplementary Fig. 1h), which mediate axonal growth control though the interaction with the type-IIa RPTPs^19^. This third interface is formed primarily by hydrophilic interactions (Fig. 1e): a positively charged cluster comprising Arg75, Arg95 and Arg98 of PTPδ forms a hydrogen bond network with a negatively charged cluster comprising Glu291 and Asp292 of IL1RAPL1. This network is stabilized by a cation-π interaction between Phe289 of IL1RAPL1 and Arg98 of PTPδ. In addition, Arg124 of PTPδ forms a hydrogen bond with Glu337 of IL1RAPL1. To confirm the importance of this interface, IL1RAPL1 Asp292–PTPδ Arg75 interacting pair was disrupted by Ala replacement. The D292A mutation of IL1RAPL1 and the R75A mutation of PTPδ reduced the affinity between IL1RAPL1 and PTPδ 15- and 19-fold, respectively (Table 3). When comparing the amino-acid sequences between IL1RAPL1 and IL-1RAcP, the Ig3 domain is the least conserved domain (23% identity) among three Ig domains. As described below, the Ig3 domain of IL-1RAcP does not interact with PTPδ in the PTPδ
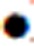
IL-1RAcP complex. Therefore, the negatively charged cluster of the IL1RAPL1 Ig3 domain and the positively charged cluster of the PTPδ Ig1 domain are another determinant for selective binding between PTPδ and IL1RAPL1.

### Structure of the complex between PTPδ and IL-1RAcP

**Figure 2:**
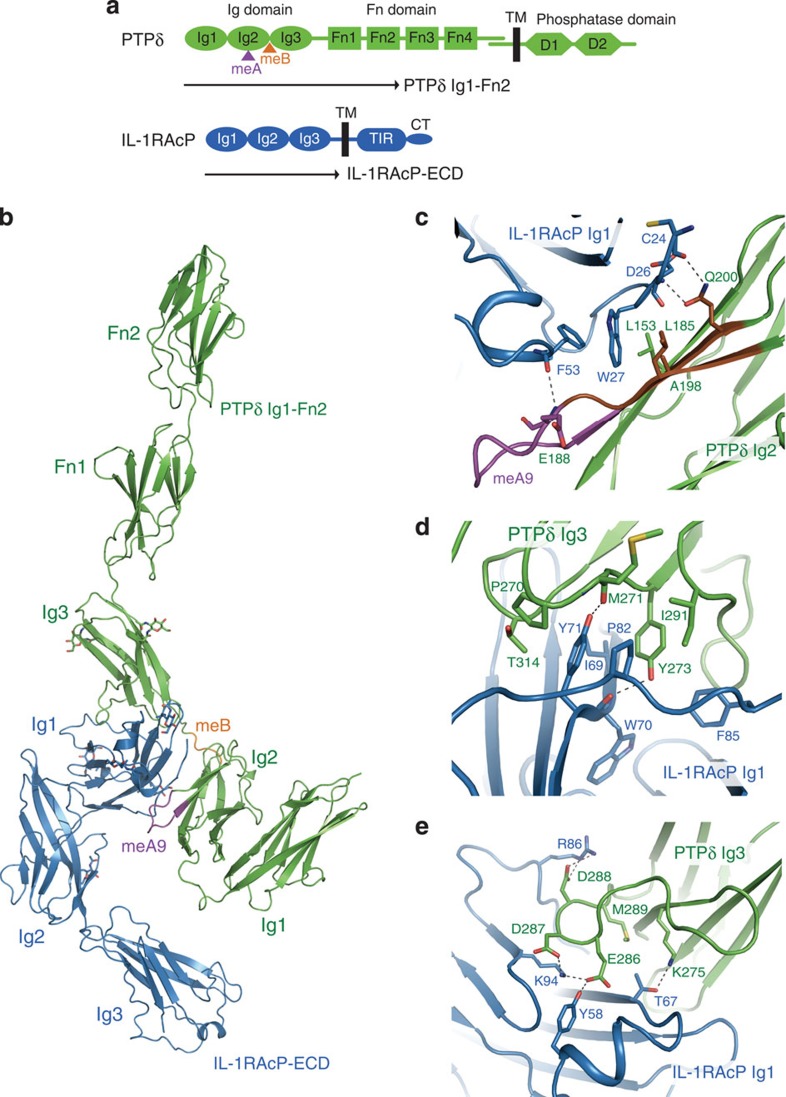
Structure of the complex between PTPδ and IL-1RAcP. (**a**) Domain organizations of PTPδ and IL-1RAcP. The meA and meB insertion sites of PTPδ are indicated by magenta and orange triangles, respectively. (**b**) Overall structure of the PTPδ Ig1–Fn2
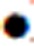
IL-1RAcP-ECD complex. PTPδ and IL-1RAcP are coloured green and blue, respectively. The meA9 and meB insertions are highlighted in magenta and orange, respectively. *N*-linked glycans are shown as sticks. (**c**) Interactions between the Ig1 domain of IL-1RAcP and the Ig2 domain of PTPδ. The colouring scheme is the same as that in **b**, except that IL1RAPL1/IL-1RAcP-Ig1-interacting β-strands of PTPδ are coloured brown. Residues involved in the interactions are shown as sticks. Hydrogen bonds are indicated by dotted lines. (**d**) Hydrophobic interactions between the Ig1 domain of IL-1RAcP and the Ig3 domain of PTPδ. The representation scheme is the same as that in **c**. (**e**) Hydrophilic interactions between the Ig1 domain of IL-1RAcP and the Ig3 domain of PTPδ. The representation scheme is the same as that in **c**.

IL-1RAcP, a member of the same IL-1R family as IL1RAPL1 (∼30% sequence identity), also functions as a postsynaptic organizer for PTPδ. However, the affinity of IL-1RAcP for PTPδ (*K*_d_: 0.68 μM) is fivefold lower than that of IL1RAPL1 (Table 2). The meA variation hardly affects the affinity. In addition, IL-1RAcP can bind to the PTPδ variant lacking both meA and meB (*K*_d_: 2.4 μM) in contrast to IL1RAPL1. To structurally explain these differences between IL1RAPL1 and IL-1RAcP, we also determined the crystal structure of IL-1RAcP-ECD in complex with PTPδ containing three Ig domains and two Fn domains (Ig1-Fn2) at 3.25 Å resolution (Fig. 2a,b, Table 1). We had confirmed that PTPδ Ig1-Fn2 efficiently binds to IL-1RAcP-ECD by testing various lengths of PTPδ (Supplementary Fig. 2a). The Ig1–Ig2 unit of PTPδ is insufficient for binding to IL-1RAcP. Similarly to the PTPδ
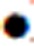
IL1RAPL1 complex, the Ig1-Fn2 domains of PTPδ bind to IL-1RAcP-ECD at a ratio of 1:1 (Fig. 2b). The asymmetric unit contains one PTPδ
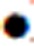
IL-1RAcP complex (Supplementary Fig. 2b). The crystal packing patterns of the PTPδ
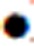
IL1RAPL1 and PTPδ
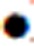
IL-1RAcP complexes in this study are totally different from each other and not suggestive of their lateral associations in synapses. Although it can be assumed that activations of PTPδ and IL1RAPL1/IL-1RAcP are mediated by a ligand-induced monomer–dimer transition by analogy with those of receptor tyrosine kinases^20^,^21^,^22^,^23^ and Toll/IL-1-receptor families^24^, our crystallographic and multiangle light scattering analyses using the ECDs of PTPδ, IL-1RAcP and IL1RAPL1 could not support this activation mechanism (Supplementary Fig. 1b, 2b,c). The Ig1-Fn2 domains of PTPδ exhibit an elongated shape with a length of 160 Å along the longest axis. Although overall shape of the IL-1RAcP-bound PTPδ Ig1-Fn2 resembles that of the IL1RAPL1-bound PTPδ Ig1-Fn2, the position and/or orientation of each domain are somewhat different between them. The Ig1–Ig3 domains of IL-1RAcP are arranged in an L-shape similar to those of IL1RAPL1, except that the relative orientation between the Ig1 and Ig2–Ig3 domains is changed by 20 degree (Supplementary Fig. 2d). Correspondingly, the Ig3 domain of IL-1RAcP does not interact with PTPδ in contrast to that of IL1RAPL1. Superposition between the Ig3 domains of IL1RAPL1 and IL-1RAcP shows that the binding surface of IL1RAPL1 for the Ig1 domain of PTPδ is not conserved in IL-1RAcP (Supplementary Fig. 2e).

### Interactions between IL-1RAcP Ig1 and PTPδ Ig2

Similarly to the PTPδ
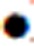
IL1RAPL1 complex, interactions between the Ig1 domain of IL-1RAcP and the Ig2 domain of PTPδ occur on the IL1RAPL1/IL-1RAcP-Ig1-interacting β-strands (Fig. 2c). The interface between the IL-1RAcP Ig1 and PTPδ Ig2 domains comprises both hydrophobic and hydrophilic interactions with a buried surface area of 726 Å^2^. Trp27 of IL-1RAcP hydrophobically interacts with Leu153, Ala198 and Leu185 of PTPδ (Fig. 2c). The W27A mutation of IL-1RAcP reduced the affinity sevenfold (Table 4). Although Arg196 of PTPδ is positioned in close proximity to Asp30 of IL-1RAcP, no observed electron density of its side chain guanidino group indicates that the interaction between Arg196 of PTPδ and Asp30 of IL-1RAcP is substantially weak (Supplementary Fig. 2f). Thus, the D30A mutation of IL-1RAcP and the R196A mutation of PTPδ hardly affected the affinity (Table 4). Basically, meA does not extensively interact with IL-1RAcP in the PTPδ Ig1-Fn2
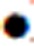
IL-1RAcP-ECD complex, consistent with the finding that the variation of meA does not affect the affinity for IL-1RAcP (Table 2). Only one meA-mediated interaction is a hydrogen bond between the main-chain N atom of PTPδ Glu188 and the main-chain O atom of IL-1RAcP Phe53, which could be formed in the shorter mini-exon variants, meA6 and meA3, but not in the variant lacking meA. The complete deletion of meA shortens the IL1RAPL1/IL-1RAcP-Ig1-interacting β-strands, which might disturb and weaken the hydrophobic interaction of IL-1RAcP Trp27 with Leu153, Ala198 and Leu185 of PTPδ, resulting in the threefold reduced affinity to IL-1RAcP (Table 2; compare meA9/+ with −/+). These structural features may reflect that the meA insertion increases the affinity between PTPδ and IL-1RAcP, independently of its variation.

### Relationship of meB with IL-1RAcP Ig1–PTPδ Ig3 interaction

The position and orientation of the PTPδ Ig3 domain relative to the IL-1RAcP Ig1 domain are similar to those relative to the IL1RAPL1 Ig1 domain (Figs 1b and 2b). The interface between the IL-1RAcP Ig1 and PTPδ Ig3 domains buries a surface area of 906 Å^2^. The inside of the interface is stabilized by more tightly packed hydrophobic interactions than that of the IL1RAPL1 Ig1–PTPδ Ig3 interface (Figs 1d and 2d). The side chains of IL-1RAcP Tyr71 and PTPδ Tyr273 are stacked in an antiparallel manner to form a hydrophobic core, which is surrounded by the side chains of IL-1RAcP Phe85, Pro82 and Ile69 and PTPδ Pro270 and Ile291 and the aliphatic portion of PTPδ Thr314. The side chains of IL-1RAcP Tyr71 and PTPδ Tyr273 are stabilized by hydrogen bonds with the main-chain O atoms of PTPδ Met271 and IL-1RAcP Trp70, respectively. Furthermore, the Ig1 domain of IL-1RAcP recognizes a negatively charged loop comprising Glu286, Asp287 and Asp288 of PTPδ. Tyr58 and Arg86 of IL-1RAcP hydrogen bond with Glu286 and Asp288 of PTPδ, respectively, whereas Lys94 of IL-1RAcP hydrogen bonds with both Glu286 and Asp287 of PTPδ (Fig. 2e). Conformation of this negatively charged loop is supported by Met289 of PTPδ. In addition, Thr67 of IL-1RAcP hydrogen bonds with Lys275 of PTPδ. Mutations of the IL-1RAcP Ig1–PTPδ Ig3 interface impaired the binding affinity more drastically than those of the IL1RAPL1 Ig1–PTPδ Ig3 interface: the Y273A mutation of PTPδ and the I69A/Y71A/P82A/F85A quadruple mutation of IL-1RAcP completely eliminated the binding ability, whereas the K94A mutation of IL-1RAcP and the E286A mutation of PTPδ decreased the affinity 12- and 8-fold, respectively (Table 4). Therefore, the interface mediated by PTPδ Ig3 is more critically important for binding to IL-1RAcP than for binding to IL1RAPL1, consistent with the finding that PTPδ Ig3 is essential for binding to IL-1RAcP but not to IL1RAPL1 (Supplementary Figs 1a and 2a).

Replacement of the native meB (Glu-Leu-Arg-Glu) by a Gly-Ser-Ser-Gly or Gln-Leu-Glu-Gln tetrapeptide hardly affected the affinity for IL-1RAcP, whereas that by two or three tandem units reduced the affinity two- or sixfold, respectively (Table 4). These impacts on the affinity for IL-1RAcP are similar to those for IL1RAPL1, supporting the notion that the meB insertion acts as an adjustable linker to control relative positions and orientations of the PTPδ Ig2 and Ig3 domains for their simultaneous interactions with the Ig1 domain of IL-1RAcP.

### Synaptogenic activity

**Figure 3:**
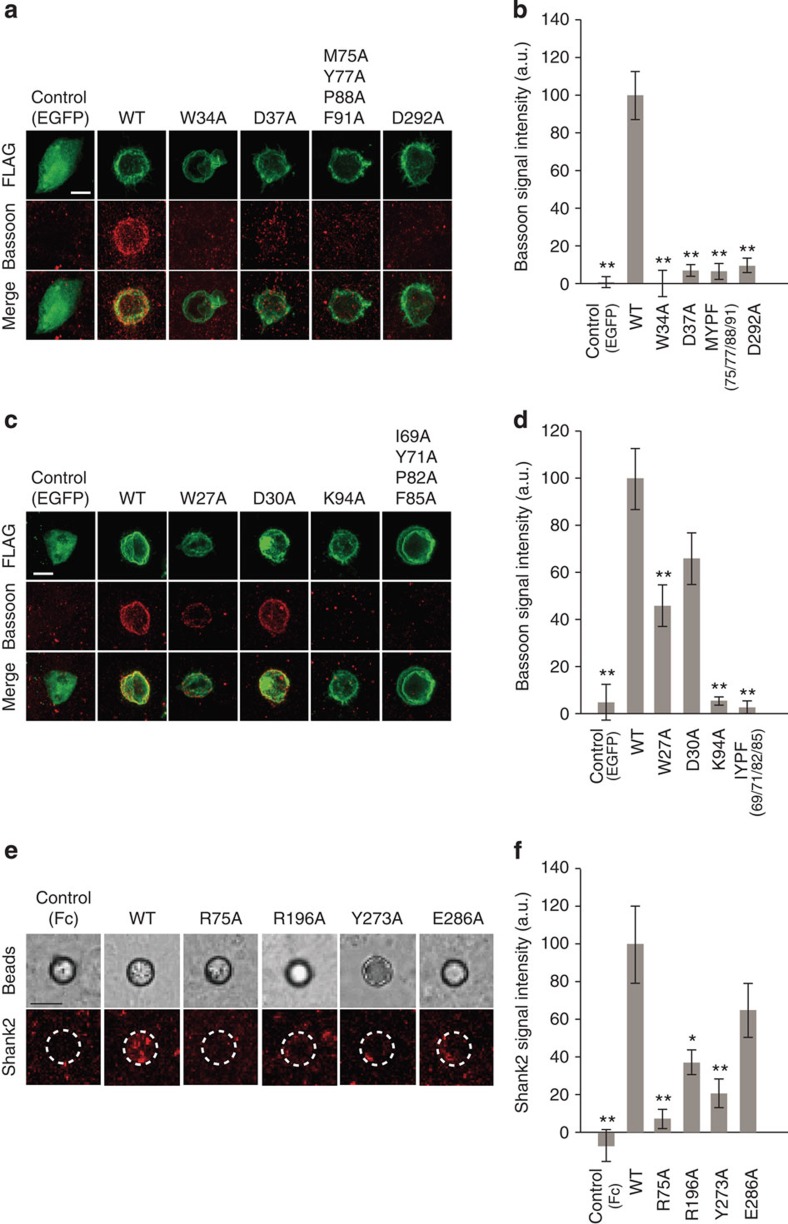
Synaptogenic activities of IL1RAPL1, IL-1RAcP and PTPδ mutants. (**a**,**c**) Accumulation of Bassoon signals (red) of cortical neurons co-cultured with HEK293T cells expressing FLAG-tagged IL1RAPL1 mutants (green) (**a**) and IL-1RAcP mutants (green) (**c**). (**b**,**d**) Intensity of staining signals for Bassoon on the surface of HEK293T cells expressing FLAG-tagged IL1RAPL1 mutants (**b**) and FLAG-tagged IL-1RAcP mutants (**d**) co-cultured with cortical neurons (*n*=16–19). (**e**) Accumulation of Shank2 signals (red) of cortical neurons co-cultured with magnetic beads conjugated with ECDs of PTPδ mutants. (**f**) Intensity of staining signals for Shank2 on the surface of magnetic beads conjugated with ECDs of PTPδ mutants co-cultured with cortical neurons (*n*=27–38). All values represent mean±s.e.m. *P<0.05 and **P<0.01 compared with HEK293T cells expressing wild-type IL1RAPL1 (**b**), wild-type IL-1RAcP (**d**) or wild-type PTPδ-ECD-Fc coated beads (**f**); Tukey's test. Scale bars, 10 μm (**a**,**c**) and 5 μm (**e**).

Finally, we investigated relationships between the structures and synaptogenic activity (that is, IL1RAPL1/IL-1RAcP-induced presynaptic differentiation and PTPδ-induced postsynaptic differentiation). IL1RAPL1/IL-1RAcP mutations were examined by fibroblast-neuron mixed culture assays (Fig. 3): HEK293T cells expressing IL1RAPL1/IL-1RAcP mutants were co-cultured with cortical neurons and then, accumulation of a presynaptic protein Bassoon in the cortical neurons was analysed (Fig. 3a–d). Because IL1RAPL1- and IL-1RAcP-induced presynaptic differentiation is completely abolished and decreased by ∼70% in cortical neurons prepared from PTPδ-deficient mice, respectively^7^,^8^, IL1RAPL1/IL-1RAcP-induced presynaptic differentiation is mostly mediated by the mixed, cognate PTPδ variants in neurons. On the other hand, to examine postsynapse-inducing activity of PTPδ mutants, magnetic beads conjugated with the ECDs of PTPδ mutants were co-cultured with cortical neurons, and accumulation of a postsynaptic protein Shank2 around the beads was analysed (Fig. 3e,f). PTPδ-stimulating postsynaptic differentiation is mediated by IL1RAPL1, IL-1RAcP and possibly other unidentified postsynaptic organizer(s) in neurons.

Synaptogenic activities of IL1RAPL1 and IL-1RAcP mutants are basically correlated to their affinities to PTPδ, which govern stimulation of the receptor activation and signal transmission inside cells. Synaptogenic activities of PTPδ mutants are also correlated with their affinities to IL1RAPL1. Remarkably, the R75A mutation in the Ig1 domain of PTPδ reduced the synaptogenic activity to ∼7%. This indicates that the interaction between the IL1RAPL1 Ig3 and PTPδ Ig1 domains is critically important for inducing postsynaptic differentiation. In contrast to the affinity of IL1RAPL1, that of IL-1RAcP is not so consistently correlated with synaptogenic activities of PTPδ mutants, possibly due to a lack of the interaction between the IL-1RAcP Ig3 and PTPδ Ig1 domains. Considering that IL-1RAcP knockout neurons retain nearly full activity of PTPδ-induced postsynaptic differentiation, we speculate that the PTPδ
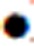
IL-1RAcP complex might function as a unidirectional organizer that is primarily responsible for inducing presynaptic differentiation *in vivo*, although IL-1RAcP can promote postsynaptic differentiation of cultured cortical neurons by binding to the meA3-containing PTPδ variant, which is specific to IL-1RAcP^7^. The Ig1 domain of PTPδ might be universally important for postsynaptic differentiation *in vivo*, which involves IL1RAPL1 and possibly other unidentified postsynaptic organizer(s).

## Discussion

Recently, crystal structures of two different *trans*-synaptic type-IIa RPTP complexes have been reported^14^,^25^. One is the complex between Ig1–Ig2 of PTPσ and leucine-rich repeat (LRR)-Ig1of TrkC^25^, while the other is that between Ig1–Ig3 of PTPδ and the first LRR (LRR1) of Slitrk1 (ref. 14, Supplementary Fig. 3a). These and our type-IIa RPTP complex structures illustrate that the type-IIa RPTPs can be recognized by structurally distinct architectures. The TrkC- or Slitrk1-interacting surface of the RPTPs is separated from the IL1RAPL1- or IL-1RAcP-interacting surface, except that a part of the TrkC-interacting surface is overlapped with the IL1RAPL1-interacting surface. The conserved arginine residues in Ig1 of the RPTPs (that is, Arg95 and Arg98 of PTPδ and Arg96 and Arg99 of PTPσ) are recognized by both IL1RAPL1 and TrkC through similar hydrogen bond interactions (Supplementary Fig. 3b). Despite the separation of these binding surfaces of the RPTPs, pair-wise superposition between any two of these complex structures shows steric clashes between the bound postsynaptic partners, ruling out their simultaneous binding to the type-IIa RPTPs (Supplementary Fig. 3c). The IL1RAPL1- or IL-1RAcP-interacting residues of PTPδ are mostly conserved in the type-IIa RPTPs. Their differences in the specificity to IL1RAPL1 or IL-1RAcP appear to be dependent exclusively on the variation of meA and meB. Our previous analysis of *Ptprs* and *Ptprf* cDNA from the developing mouse brain suggested that none of the PTPσ variants contains the mini-exon peptide insertion at the meA site^8^ and that 90% of LAR lacks the peptide insertion at the meB site^8^. Therefore, IL1RAPL1 and IL-1RAcP induce synaptogenesis specifically through PTPδ, at least in the developing brain. On the other hand, the meB insertion is sufficient for binding to Slitrks. They may regulate synaptogenesis mainly through PTPσ or PTPδ in the developing brain^13^,^14^, although they can potentially bind to all the type-IIa RPTP variants containing meB. Both the regulation of meA and meB choice by alternative splicing of the type-IIa RPTPs and the mutually exclusive binding among postsynaptic ligands for the type-IIa RPTPs may contribute to sharpening target specificity of central synaptogenesis.

**Figure 4:**
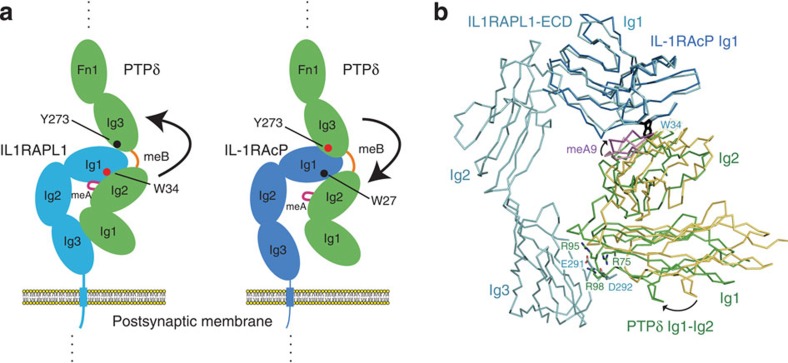
Mechanistic insights into *trans*-synaptic adhesion between PTPδ and IL1RAPL1 or IL-1RAcP. (**a**) Schematic drawing of the mechanism for the meA- and meB-regulated binding of PTPδ to IL1RAPL1 or IL-1RAcP. The colouring scheme is the same as those in Figs 1a and 2a. Red dots indicate the primary interaction site, whereas black dots indicate the secondary interaction site. Arrows indicate a model that the interaction at the primary site may be followed by the interaction at the secondary site. (**b**) Superposition of the Ig1–Ig2 unit in the IL-1RAcP-bound PTPδ (yellow) on that in the IL1RAPL1-bound PTPδ (green), using the Ig1 domain of IL1RAPL1/IL-1RAcP as the reference. Residues involved in the IL1RAPL1 Ig3–PTPδ Ig1 interface and the corresponding IL-1RAcP residues are shown as sticks.

The Ig–Ig interaction between cell-surface receptors is generally important for cell adhesion and communication in immune and neuronal systems. To our knowledge, binding between Ig-containing adhesion receptors is typically mediated by single, homotypic Ig–Ig interactions^26^,^27^,^28^ in neuronal systems. In contrast, binding between PTPδ and IL1RAPL1/IL-1RAcP is mediated by multiple, heterotypic Ig–Ig interactions. The meB insertion of PTPδ substantially contributes to these multiple, heterotypic Ig–Ig interactions; meB is located at the junction between the Ig2 and Ig3 domain of PTPδ and adjusts their relative spacing and orientation so that they can simultaneously interact with the Ig1 domain of IL-1RAcP or IL1RAPL1 (Fig. 4a). In the IL-1RAcP/IL1RAPL1-bound state, the Ig2 and Ig3 domains of PTPδ exhibits a unique conformation that differs from a linear or V-shaped conformation, which is typically observed in a tandem repeat of Ig domains. Furthermore, this unique conformation of PTPδ Ig2–Ig3 also differs from the conformation in a Slitrk-bound state^14^. Most PTPδ variants expressed in the developing mouse brain at postnatal day 11 contain meB and only 4% of variants lack meB^8^, suggesting that the meB-containing PTPδ variants should be mostly utilized for synaptogenesis in the brain. Therefore, the insertion of meB might contribute to allowing PTPδ Ig2–Ig3 to form some distinct conformations, depending on the structurally different postsynaptic ligands.

For binding of PTPδ to IL1RAPL1, either meA9 or meA6 is essential. Thus, the interface including meA is likely the primary interaction site. Accordingly, the W34A mutation of IL1RAPL1 (at the interface with PTPδ Ig2) eliminated binding to PTPδ. Moreover, the interaction with meA appears coupled to the third interface involving PTPδ Ig1 and IL1RAPL1 Ig3, which is critical for binding between IL1RAPL1 and PTPδ and inducing bidirectional synaptic differentiation of cultured cortical neurons, as mentioned above. Comparison between the IL1RAPL1 and IL-1RAcP complexes exhibits a rotational difference in the orientations of the PTPδ Ig1–Ig2 unit, relative to the Ig1 domains of IL1RAPL1 and IL-1RAcP (Fig. 4b). Trp34 of IL1RAPL1 is positioned at the pivot for this rotation, which allows PTPδ Ig1 to interact with IL1RAPL1 Ig3. In this context, meA also contributes to the multiple, heterotypic Ig–Ig interactions between IL1RAPL1 and PTPδ, besides meB, which assists in locating the Ig3 domain of PTPδ in the appropriate position for interacting with the Ig1 domain of IL1RAPL1. Consistent with this, the impact of the lack of meB on binding between PTPδ and IL1RAPL1 is comparable to that of the PTPδ Y273A mutation (deficient in the PTPδ Ig3–IL1RAPL1 Ig1 interaction). In contrast, the impact of the lack of meB on binding between PTPδ and IL-1RAcP is incompatible to that of the PTPδ Y273A mutation, but rather is comparable to that of mutations at the IL-1RAcP Ig1–PTPδ Ig2 interface. The W27A mutation of IL-1RAcP, which is equivalent to the W34A mutation of IL1RAPL1, reduced the affinity sevenfold but did not eliminate the binding. Therefore, for binding to IL-1RAcP, the PTPδ Ig3–IL-1RAcP Ig1 interface is likely the primary interaction site, and the meB insertion may serve as the adjustable linker to locate the Ig2 domain of PTPδ in the appropriate position for interacting with the Ig1 domain of IL-1RAcP (Fig. 4a).

IL-1RAcP functions in both immune and neuronal systems, whereas IL1RAPL1 is exclusively expressed in neuronal systems, suggesting that IL1RAPL1 has evolved to be more specialized for neuronal systems than IL-1RAcP. Correspondingly, IL-1RAcP binds to PTPδ weaker than IL1RAPL1 but has a broader specificity to the meA variants. In contrast, IL1RAPL1 has more strict specificity to meA and can bind to PTPδ more tightly than IL-1RAcP. The affinity difference generated by the splice-insert signaling codes is substantially high for binding between PTPδ and IL1RAPL1 but modest for binding between PTPδ and IL-1RAcP. On the other hand, in our synaptogenic assays, the strength of the induced synaptic differentiation seems non-linearly correlated with the affinity; rather, there seems to be the threshold of the affinity for inducing the synaptic differentiation, which might contribute to controlling appropriate synaptic target selection to avoid misconnections to target cells with low affinity ligands. Further functional studies including identification and characterization of additional regulatory factors are awaited to discuss the activation and signaling mechanisms of IL1RAPL1/IL-1RAcP and PTPδ for inducing synaptic differentiation.

## Methods

### Protein expression and purification

Genes encoding mouse PTPδ-ECD, PTPδ Ig1-Fn4, PTPδ Ig1-Fn3, PTPδ Ig1-Fn2, PTPδ Ig1-Fn1, PTPδ Ig1–Ig3 and PTPδ Ig1–Ig2 (residues 28–861, 28–699, 28–611, 28–518, 28–418, 28–325 and 28–237, respectively) were amplified from cDNA (accession No. NM_011211.3) by PCR and cloned into pEBMulti-Neo vector (Wako Pure Chemical Industries) with N-terminal signal sequences derived from pHLsec vector and C-terminal hexahistidine tag^29^. PTPδA3B^−^ Ig1-Fn2 gene was also cloned from cDNA^8^ in the same manner as the PTPδ constructs. Genes encoding mouse IL-1RAcP-ECD (residues 21–351, gene accession No. NM_008364.2) and IL1RAPL1-ECD (residues 19–352, gene accession No. NM_001160403.1) were cloned into pEBMulti-Neo vector, with N-terminal Igκ signal sequence and C-terminal hexahistidine tag^7^,^8^. All proteins were transiently expressed using Freestyle 293-F cells (Invitrogen). For crystallography, proteins were purified from 0.2 to 1 l of culture media by Ni-NTA (Qiagen) or Talon metal affinity resin (Clontech) with a standard protocol and dialysed against 20 mM Tris-HCl buffer (pH 7.5) containing 150 mM NaCl. Proteins were concentrated at 5–10 gl^−1^, flash-frozen in liquid N_2_ and stored at −80 °C until use. A gene encoding PTPδ (Fn1-Fn2) (residues 328–518) was cloned into pET21a vector (Novagen). PTPδ (Fn1–Fn2) was overexpressed in Rosetta (DE3) *Escherichia coli* cells (Novagen) and purified by the Ni affinity chromatography followed by the size exclusion chromatography with Superdex 200 16/60 (GE Healthcare).

### Pull-down assay

Binding abilities of serially deleted PTPδ-ECDs were tested by pull-down assay with the C-terminally Fc-tagged IL-1RAcP- or IL1RAPL1-ECD (IL-1RAcP- or IL1RAPL1-Fc, respectively). IL-1RAcP- and IL1RAPL1-Fc were purified by Protein G Sepharose (GE Healthcare)^7^,^8^. The purified IL-1RAcP- or IL1RAPL1-Fc was mixed with the PTPδ proteins at 1:1 molar ratio and then immobilized with Protein G Sepharose beads (GE Healthcare). After washing by phosphate-buffered saline (PBS), the bound protein complexes were eluted by SDS sample loading buffer, followed by SDS–PAGE analyses.

### Crystallization

PTPδA3B^−^Ig1-Fn2, PTPδ Fn1–Fn2, IL1RAPL1 and the PTPδ Ig1–Fn2
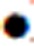
IL-1RAcP-ECD, PTPδ-ECD
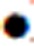
IL1RAPL1-ECD and PTPδ Ig1–Ig2
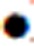
IL1RAPL1-ECD complexes were crystallized by the sitting drop vapour diffusion method. Protein solutions were mixed with the following reservoir solutions: 10% polyethylene glycol (PEG) 3350, 0.1 M ammonium iodide for PTPδA3B^−^ Ig1–Fn2; 20% PEG3350, 0.1 M MgCl_2_ and 0.1 M BisTris (pH 5.5) for PTPδ Fn1–Fn2; 2.0–2.4 M ammonium sulfate for IL1RAPL1; 15% PEG3350, 0.1 M ammonium sulfate and 0.1 M tri-sodium citrate (pH 5.5) for the PTPδ Ig1–Fn2
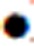
IL-1RAcP-ECD complex; 12% PEG4000, 0.1 M lithium sulfate, 0.1 M ADA (pH 6.5) for the PTPδ-ECD
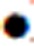
IL1RAPL1-ECD complex; 15% PEG4000, 0.1 M MES (pH 6.0) for the PTPδ Ig1–Ig2
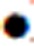
IL1RAPL1-ECD complex. For the crystallization of the complexes, two protein samples were mixed at a molar ratio of 1:1 to a final concentration of ∼6 g l^−1^ before crystallization experiments. All crystallization experiments except for PTPδ Fn1–Fn2 were performed at 20 °C. Temperature shift from 4 to 28 °C drastically improved the crystal size of PTPδ Fn1–Fn2. Crystals were flash-frozen in liquid N_2_, followed by soaking in the reservoir solutions supplemented with the following cryo-protectants: 25% glycerol for PTPδA3B^−^ Ig1–Fn2 and the PTPδ Ig1–Ig2
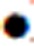
IL1RAPL1-ECD complex; 25% ethylene glycol for PTPδ Fn1–Fn2; 27% ethylene glycol for IL1RAPL1; 35% xylitol for the PTPδ Ig1–Fn2
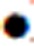
IL-1RAcP-ECD complex; 20% ethylene glycol for the PTPδ-ECD
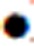
IL1RAPL1-ECD complex.

### Crystallography

All data were collected at 100 K at BL41XU in SPring-8, and processed with HKL2000 (ref. 30) and CCP4 program suite^31^. Data collection and refinement statistics were summarized in Table 1. Resolutions were estimated, basically based on *I*/*σI* values (∼2). To improve the quality of the atomic models, the resolutions of PTPδ-ECD
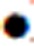
IL1RAPL1-ECD and PTPδA3B^−^ Ig1–Fn2 were set to be as high as possible. The resolution of PTPδ Fn1–Fn2 was limited by the size of the detector. We first determined a crystal structure of the PTPδ Ig1–Ig2
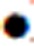
IL1RAPL1-ECD complex by the molecular replacement method using IL-1RAcP-ECD (PDB:4DEP, 3O4O)^32^,^33^ and PTPδ Ig1–Ig2 (PDB:2YD6)^19^ as the search models with the program Molrep^34^. Model building and refinement were carried out using the programs Coot^35^ and Phenix^36^, respectively. The final model of the PTPδ Ig1–Ig2
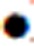
IL1RAPL1-ECD complex was refined at 2.7 Å to *R*_work_ and *R*_free_ values of 22.6 and 26.3%, respectively. Then, the PTPδ-ECD
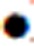
IL1RAPL1-ECD complex structure was determined by the molecular replacement method using IL1RAPL1-ECD in the PTPδ Ig1–Ig2-bound state, PTPσ Ig3 (PDB:2YD9)^19^, LAR Fn2 (PDB:2DJU) and PTPδ Fn2 (PDB:2DLH) as the search models. The final model of the PTPδ-ECD
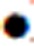
IL1RAPL1-ECD complex was refined at 4.4 Å to *R*_work_ and *R*_free_ values of 29.1 and 32.7%, respectively. In addition, 2.0-Å-resolution structure of PTPδ Fn1–Fn2 was solved by the molecular replacement method and refined with the program Phenix to *R*_work_ and *R*_free_ values of 20.4 and 22.3%, respectively. The structure of IL1RAPL1-ECD was determined by the molecular replacement method and refined at 3.0 Å to *R*_work_ and *R*_free_ values of 26.4 and 29.8%, respectively. The structure of PTPδ Ig1–Fn2
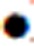
IL-1RAcP-ECD complex was determined by the molecular replacement method using IL-1RAcP-ECD (PDB:4DEP, 3O4O)^32^,^33^, PTPδ Ig1–Fn2 (PDB:2YD6)^19^ and our refined PTPδ Fn1–Fn2 structure as the search models. The final model of the PTPδ Ig1–Fn2
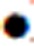
IL-1RAcP-ECD complex was refined at 3.25 Å to *R*_work_ and *R*_free_ values of 25.6 and 28.7%, respectively. The structure of PTPδA3B^−^ Ig1–Fn2 was determined by the molecular replacement method and refined at 3.5 Å to *R*_work_ and *R*_free_ values of 29.1 and 31.5%, respectively. The buried surface area was calculated with the program PISA^37^. The stereochemistry of the final model was assessed by the program Procheck. Percentages of residues in the most favoured regions of the Ramachandran plot are 87.9%, 81.2%, 86.7%, 89.4%, 88.1% and 89.1% for PTPδ Ig1–Ig2
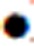
IL1RAPL1-ECD, PTPδ-ECD
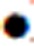
IL1RAPL1-ECD, PTPδ Fn1–Fn2, PTPδA3B^−^ Ig1–Fn2, PTPδ Ig1–Fn2
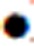
IL-1RAcP-ECD and IL1RAPL1-ECD, respectively. No residues are in the disallowed regions for all these structures. Structural figures were prepared with the program PyMol (Schrödinger, LLC).

### SPR analysis

SPR equilibrium experiments were carried out by using Biacore T200 (GE Healthcare) at 25 °C in 10 mM Hepes (pH 7.9) containing 150 mM NaCl, 0.05% Tween-20. IL1RAPL1-ECD-His_6_ was immobilized on a CM5 sensor chip by the amine-coupling method at a density of about 2,000 resonance units (RU), whereas IL-1RAcP-ECD-Fc was captured at a density of about 700 RU by anti-Fc antibodies that were immobilized on a CM5 sensor chip. Splicing variants or mutants of PTPδ Ig1–Fn1 were injected as the analytes with the concentration range from 15.6 to 4,000 nM. The IL1RAPL1-immobilized sensor chip was regenerated by 10 mM NaOH. The sensorgrams are shown in Supplementary Fig. 4.

### Cell cultures and co-culture assay

A central nervous system-restricted isoform of IL-1RAcP, IL-1RAcPb, was used for HEK293T cell-based analyses of IL-1RAcP mutants. Primary cortical cultures were prepared from mice at E18 (ref. 38). The cortical cells were placed on coverslips coated with 30 μg ml^−1^ poly-L-lysine and 10 μg ml^−1^ mouse laminin at the density of 5 × 10^5^ cells per well for co-culture assay. The cells were cultured in Neurobasal-A supplemented with 2% B-27 supplement (Invitrogen), 5% FCS, 100 U ml^−1^ penicillin, 100 μg ml^−1^ streptomycin and 0.5 mM L-glutamine for 24 h, and then cultured in the same medium without FCS. Cultures of HEK293T cells were maintained in DMEM supplemented with 10% FCS^38^. Expression vectors for mutated forms of PTPδ-ECD-Fc, FLAG-tagged IL-1RAcPb and FLAG-tagged IL1RAPL1 were generated by PCR-based mutagenesis using pEB6-PTPδ-ECD-Fc, pFLAG-IL-1RAcPb and pFLAG-IL1RAPL1 (ref. 7) as templates, respectively. Expression vectors for FLAG-tagged IL1RAPL1 and IL-1RAcP mutants were transfected to HEK293T cells using NanoJuice transfection reagent (Novagen). After 2 days of culture, the transfected cells were washed with PBS containing 2 mM EDTA, and incubated with the same buffer at 37 °C for 10 min. Dispersed cells were plated onto cortical neurons at days *in vitro* (DIV) 8. After 24 h of co-culture, cells were fixed for immunostaining with mouse anti-Bassoon (Stressgen, 1:300) and rabbit anti-FLAG (Sigma, 1:1,000) antibodies. Fc and ECDs of PTPδ mutant proteins fused to Fc in HEK293T cell culture medium were bound to Protein A-conjugated magnetic particles (smooth surface, 4.0–4.5 μm diameter; Spherotech). Beads coupled with Fc or Fc fusion proteins were added to cortical neurons (DIV 14). After 24 h, cultures were fixed for immunostaining with rabbit anti-Shank2 antibody (Frontier Institute, 1:200).

### Image acquisition and quantification

Images of fibroblast–neuron or bead–neuron co-culture experiments were acquired with a confocal laser-scanning microscope TCS SP5II (Leica) under constant conditions as to laser power, iris, gain, *z*-steps and zoom setting throughout the experiments. The images were collected from at least two separate cell cultures. All quantitative measurements were performed with ImageJ 1.36b software^39^. The intensities of immunostaining signals for Bassoon or Shank2 were measured as the optical mean density within circles of 30- and 7-μm diameters enclosing transfected HEK293T cells and coated-beads, respectively. Statistical significance was evaluated by one-way analysis of variance followed by *post hoc* Tukey's test.

## Author contributions

T.Y., A.M., T.S. and A.Y. performed pull-down assays and crystallization. A.Y., S.F., Y.S. and S.G.-I. collected diffraction data. A.Y. and S.F. analysed the collected data and determined the structures. T.Y., T.S. and S.I.-O. performed cell biological experiments. A.Y., T.Y. and S.F. wrote the paper with editing by T.U., H.M. and M.M. T.Y., M.M. and S.F. designed and supervised the study.

## Additional information

**Accession codes:** Atomic coordinates and structure factors have been deposited with the accession codes 4YFC (PTPδ Ig1–Ig2
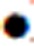
IL1RAPL1-ECD), 4YFD (PTPδ Ig1–Fn2
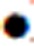
IL-1RAcP-ECD), 4YFE (PTPδ Fn1–Fn2), 4YFG (PTPδA3B^−^ Ig1–Fn2), 4YH6 (IL1RAPL1-ECD) and 4YH7 (PTPδ-ECD
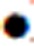
IL1RAPL1-ECD).

**How to cite this article:** Yamagata, A. *et al*. Mechanisms of splicing-dependent *trans*-synaptic adhesion by PTPδ–IL1RAPL1/IL-1RAcP for synaptic differentiation. *Nat. Commun*. 6:6926 doi: 10.1038/ncomms7926 (2015).

## Supplementary Material

Supplementary Information Supplementary Figures 1-4

## Figures and Tables

**Table 1:** Data collection and refinement statistics.

	PTPδ Ig1–Ig2 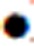 IL1RAPL1-ECD	PTPδ-ECD 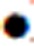 IL1RAPL1-ECD	PTPδ Fn1–Fn2	PTPδA3B^−^Ig1–Fn2	PTPδ Ig1–Fn2 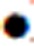 IL-1RAcP-ECD	IL1RAPL1-ECD
*Data collection*						
Space group	*C*2	*I*4_1_	*P*2_1_2_1_2_1_	*P*1	*P*2	*P*4_2_2_1_2
Cell dimensions						
*a*, *b*, *c* (Å)	163.0, 81.2, 91.5	286.3, 286.3, 70.2	51.9, 65.1, 134.0	70.5, 72.6, 94.4	77.5, 63.3, 169.4	102.5, 102.5, 222.7
α, β, γ (°)	90.0, 91.2, 90.0	90.0, 90.0, 90.0	90.0, 90.0, 90.0	107.6, 94.4, 108.5	90.0, 94.2, 90.0	90.0, 90.0, 90.0
Resolution (Å)	50–2.70 (2.75–2.70)	50–4.40 (4.48–4.40)	50.0–1.97 (2.00–1.97)	50.0–3.50 (3.56–3.50)	50.0–3.25 (3.31–3.25)	50–3.00 (3.05–3.00)
*R*_sym_	0.12 (0.43)	0.07 (0.42)	0.12 (0.33)	0.19 (0.49)	0.12 (0.44)	0.12 (0.38)
*I*/*σI*	12.2 (2.0)	17.7 (1.7)	26.6 (4.4)	4.8 (1.6)	10.7 (2.0)	10.3 (2.3)
Completeness (%)	98.7 (95.9)	95.6 (86.0)	99.8 (99.7)	91.4 (84.2)	97.3 (92.5)	97.5 (93.4)
Redundancy	5.1 (3.2)	4.2 (2.2)	10.7 (7.7)	2.5 (1.9)	4.7 (2.9)	7.1 (3.3)
						
*Refinement*						
Resolution (Å)	2.70	4.4	1.97	3.50	3.25	3.00
No. reflections	32,256	17,618	32,731	18,642	25,436	23,998
*R*_work_/*R*_free_	0.226/0.263	0.291/0.327	0.204/0.223	0.291/0.315	0.256/0.287	0.264/0.298
No. of atoms						
Protein	4,208	7,124	2,988	7,412	6,407	4,965
Sugar	143	117		56	98	325
Water	77		308			
*B*-factors (Å^2^)						
Protein	66.90	147.10	28.90	138.50	107.90	64.70
Sugar	106.50	147.90		147.80	121.10	96.80
Water	62.90		34.70			
R.m.s. deviations						
Bond lengths (Å)	0.005	0.006	0.006	0.005	0.005	0.004
Bond angles (°)	1.28	1.55	1.32	1.15	1.07	0.81

Values in parentheses are for highest-resolution shell.

**Table 2:** Affinities (*K*_D_) between the splicing variants of PTPδ and IL1RAPL1/IL-1RAcP.

PTPδ (meA/meB)	IL1RAPL1 (μM)	IL-1RAcP (μM)
meA9/+	0.15±0.011	0.68±0.013
meA9/−	0.80±0.11	2.0±0.085
meA6/+	0.81±0.14	0.73±0.013
meA6/−	ND	2.3±0.016
meA3/+	ND	0.51±0.010
meA3/−	ND	2.8±0.053
−/+	ND	2.2±0.075
−/−	ND	2.4±0.075

ND, not detectable.

Data are presented as mean±s.d.

**Table 3:** Affinities (*K*_D_) of PTPδ or IL1RAPL1 mutants for binding between PTPδ and IL1RAPL1.

	IL1RAPL1 (μM)
*PTPδ*	
WT	0.15±0.011
R75A	2.8±0.34
R196A	1.7±0.45
Y273A	0.86±0.11
meB	
GSSG	0.14±0.022
QLEQ	0.16±0.0026
ELREELRE	0.15±0.052
ELREELREELRE	0.42±0.038
	
	**PTPδ (μM)**
*IL1RAPL1*	
WT	0.15±0.011
W34A	ND
D37A	2.4±0.016
M75A/Y77A/P88A/F91A	2.8±0.48
D292A	2.2±0.075

ND, not detectable; WT, wild type.

Data are presented as mean±s.d.

**Table 4:** Affinities (*K*_D_) of PTP**δ** or IL-1RAcP mutants for binding between PTP**δ** and IL-1RAcP.

	IL-1RAcP (μM)
*PTPδ*	
WT	0.68±0.013
R75A	1.1±0.023
R196A	0.89±0.17
Y273A	ND
E286A	5.3±0.52
meB	
GSSG	0.64±0.036
QLEQ	0.91±0.019
ELREELRE	1.5±0.038
ELREELREELRE	3.8±0.21
	
	**PTPδ (μM)**
*IL-1RAcP*	
WT	0.68±0.013
W27A	4.6±0.076
D30A	0.62±0.033
K94A	8.2±0.054
I69A/Y71A/P82A/F85A	ND

ND, not detectable; WT, wild type.

Data are presented as mean±s.d.
