# Supplementary material for: Mechanisms of splicing-dependent trans-synaptic adhesion by PTPδ–IL1RAPL1/IL-1RAcP for synaptic differentiation
Source: Nat Commun. 2015 Apr 24;6:6926. doi: 10.1038/ncomms7926 (PMC4423211; doi:10.1038/ncomms7926)
Supplement: Supplementary Information — Supplementary Figures 1-4 [file ncomms7926-s1.pdf]

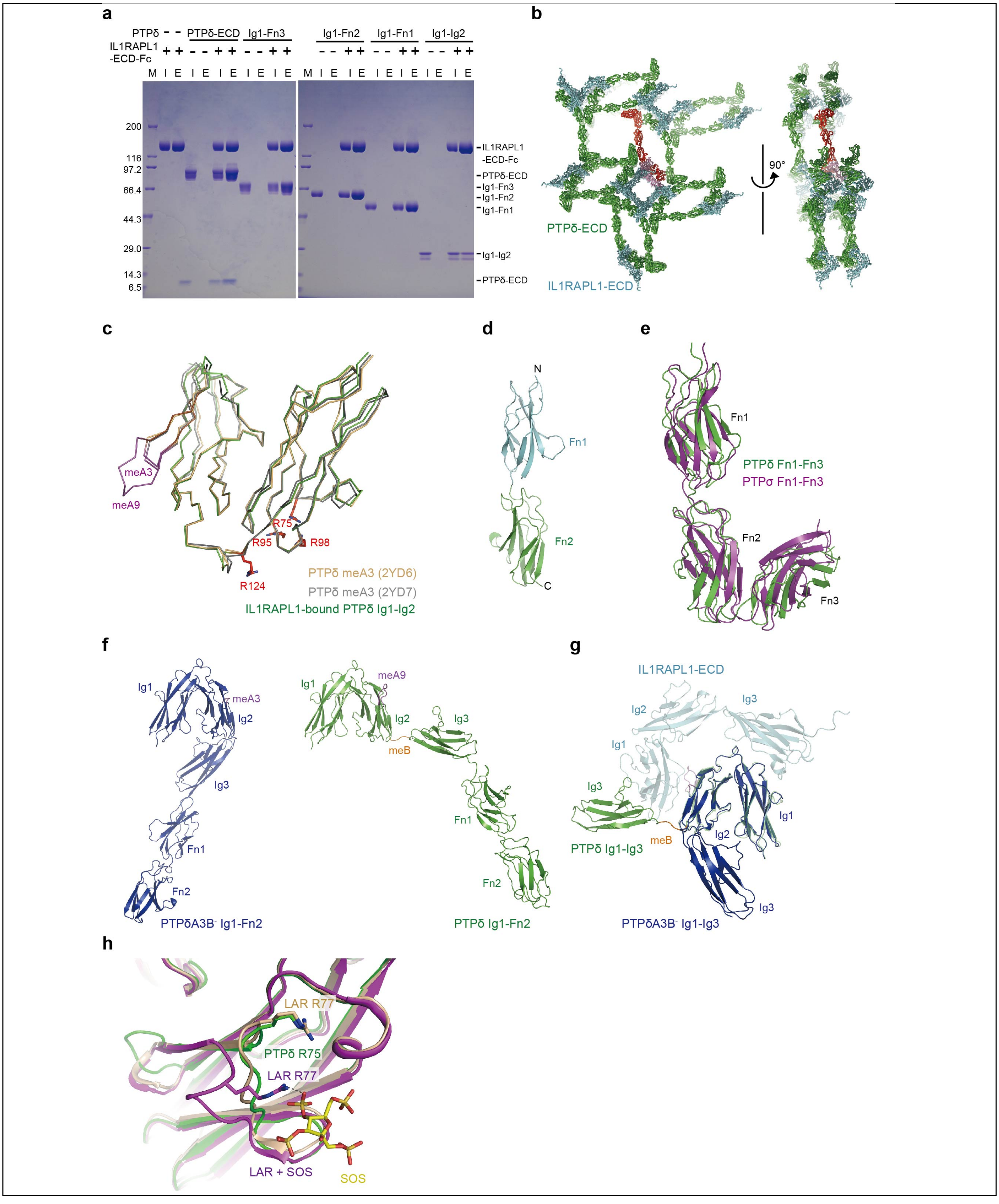

a
PTPδ
IL1RAPL1 -ECD-Fc
PTPδ-ECD
Ig1-Fn3
Ig1-Fn2
Ig1-Fn1
Ig1-Ig2
M I E I E I E I E I E
200
116
97.2
66.4
44.3
29.0
14.3
6.5
IL1RAPL1 -ECD-Fc
PTPδ-ECD
Ig1-Fn3
Ig1-Fn2
Ig1-Fn1
Ig1-Ig2
PTPδ-ECD
b
90°
PTPδ-ECD
IL1RAPL1-ECD
c
meA3
meA9
R75
R95
R98
R124
PTPδ meA3 (2YD6)
PTPδ meA3 (2YD7)
IL1RAPL1-bound PTPδ Ig1-Ig2
d
N
Fn1
Fn2
C
e
Fn1
PTPδ Fn1-Fn3
PTPσ Fn1-Fn3
Fn2
Fn3
f
Ig1
meA3
Ig2
Ig3
Fn1
Fn2
PTPδA3B⁻ Ig1-Fn2
Ig1
meA9
Ig2
Ig3
meB
Fn1
Fn2
PTPδ Ig1-Fn2
g
IL1RAPL1-ECD
Ig2
Ig3
Ig1
Ig3
PTPδ Ig1-Ig3
meB
Ig1
Ig2
Ig3
PTPδA3B⁻ Ig1-Ig3
h
LAR R77
PTPδ R75
LAR R77
LAR + SOS
SOS

Supplementary Figure 1

Functional and structural characterization of the PTPδ•IL1RAPL1 complex

(a) Serial deletion analyses of PTPδ-ECD for binding to IL1RAPL1-ECD. IL1RAPL1-bound PTPδ proteins were resolved by SDS-PAGE without boiling and stained by Coomassie Brilliant Blue. IL1RAPL1-Fc migrated as an Fc-mediated dimer. Lanes I, E and M indicate the input, elution and molecular weight marker, respectively. Note that the C-terminal region of PTPδ-ECD (corresponding to the lower-molecular-weight band) is non-covalently attached to the rest of PTPδ-ECD (corresponding to the higher-molecular-weight band).

(b) Crystal packing of the PTPδ-ECD•IL1RAPL1-ECD complex. PTPδ-ECD and IL1RAPL1 in one complex are highlighted in red and magenta, respectively, whereas those in the crystallographic symmetry-related complexes are colored green and cyan, respectively.

(c) Superposition of the IL1RAPL1-bound PTPδ Ig1-Ig2 on the isolated PTPδA3 Ig1-Ig2 (PDB:2YD6 and 2YD7).

(d) Crystal structure of the isolated PTPδ Fn1-Fn2 at 2.0 Å resolution. The Fn1 and Fn2 domains are colored cyan and green, respectively.

(e) Superposition of the IL1RAPL1-bound PTPδ Fn1-Fn3 (green) on the apo PTPσ Fn1-Fn3 (magenta) (PDB:4PBX), using the Fn1-Fn2 unit as the reference.

(f) Ig1-Fn2 structures of the apo PTPδA3B$^-$ and the IL1RAPL1-bound PTPδ Ig1-Fn2.

(g) Superposition of the apo PTPδA3B$^-$ on the IL1RAPL1-bound PTPδ, using the Ig1-Ig2 unit as the reference.

(h) Superposition of the Ig1 domain in the IL1RAPL1-bound PTPδ (green) on those in the apo (light brown) and SOS-bound LARs (magenta). SOS, Arg75 of PTPδ and Arg77 of LAR are shown as sticks.

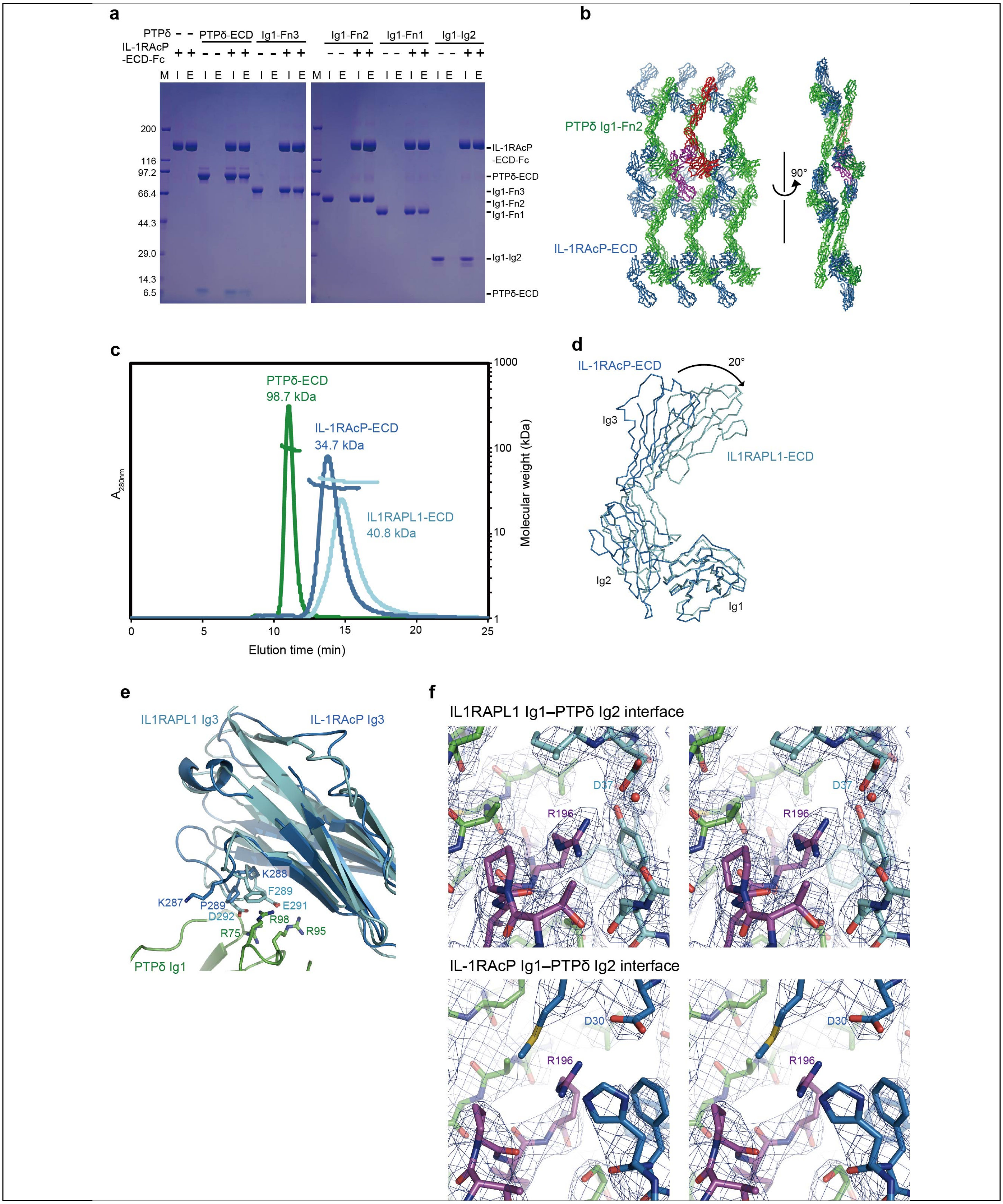

a
PTPδ
IL-1RAcP -ECD-Fc
PTPδ-ECD
Ig1-Fn3
Ig1-Fn2
Ig1-Fn1
Ig1-Ig2
M I E I E I E I E I E M I E I E I E I E I E I E
200
116
97.2
66.4
44.3
29.0
14.3
6.5
IL-1RAcP -ECD-Fc
PTPδ-ECD
Ig1-Fn3
Ig1-Fn2
Ig1-Fn1
Ig1-Ig2
PTPδ-ECD
b
PTPδ Ig1-Fn2
IL-1RAcP-ECD
90°
c
PTPδ-ECD 98.7 kDa
IL-1RAcP-ECD 34.7 kDa
IL1RAPL1-ECD 40.8 kDa
A280nm
Molecular weight (kDa)
Elution time (min)
d
IL-1RAcP-ECD
20°
Ig3
IL1RAPL1-ECD
Ig2
Ig1
e
IL1RAPL1 Ig3
IL-1RAcP Ig3
K288
F289
K287
P289
E291
D292
R98
R75
R95
PTPδ Ig1
f
IL1RAPL1 Ig1–PTPδ Ig2 interface
D37
R196
IL-1RAcP Ig1–PTPδ Ig2 interface
D30
R196

Supplementary Figure 2

Functional and structural characterization of PTPδ•IL-1RAcP complex

(a) Serial deletion analyses of PTPδ-ECD for binding to IL-1RAcP-ECD. IL-1RAcP-bound PTPδ proteins were resolved by SDS-PAGE without boiling and stained by Coomassie Brilliant Blue. IL-1RcAP-Fc migrated as an Fc-mediated dimer. The presentation scheme is the same as that in Supplementary Fig. 1a.

(b) Crystal packing of the PTPδ Ig1-Fn2•IL-1RAcP-ECD complex. PTPδ Ig1-Fn2 and IL-1RAcP in one complex are highlighted in red and magenta, respectively, whereas those in the crystallographic symmetry-related complexes are colored green and cyan, respectively.

(c) SEC-MALS analyses of IL-1RAcP-, IL1RAPL1- and PTPδ-ECDs (colored blue, cyan and green, respectively). Chromatograms and calculated molecular weights are shown.

(d) Superposition of the PTPδ-bound IL-1RAcP (blue) on the PTPδ-bound IL1RAPL1 (cyan), using the Ig1 domain of IL-1RAcP/IL1RAPL1 as the reference.

(e) Structural alignment of the Ig3 domains between IL1RAPL1 and IL-1RAcP. The Ig3 domain of IL-1RAcP (blue) is superposed on that of IL1RAPL1 (cyan).

(f) $2F_o$-F_c electron density map around Arg196 of PTPδ in the complex with IL1RAPL1 and that in the complex with IL-1RAcP, contoured at 1.0 σ level (stereo view).

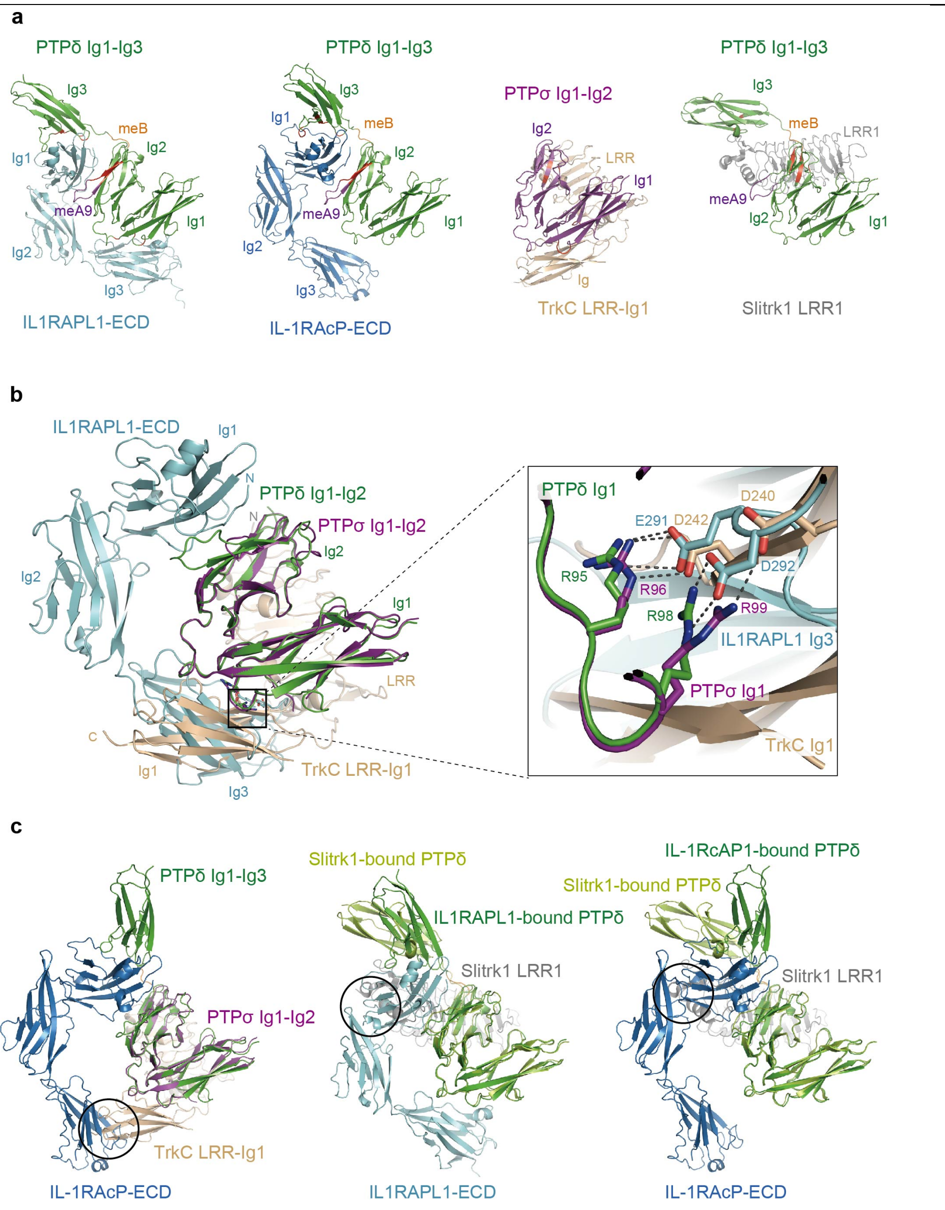

Supplementary Figure 3

Structural comparison of *trans*-synaptic type-IIa RPTP complexes

(a) Structures of the IL1RAPL1•PTPδ-ECD, IL-1RAcP•PTPδ Ig1-Fn2, TrkC LRR-Ig1•PTPσ Ig1-Ig2, Slitrk1 LRR1•PTPδ Ig1-Ig3 complexes. Fn domains are omitted for clarity. Binding interfaces of the RPTPs for postsynaptic partners are highlighted in red.

(b) Superposition of the TrkC LRR-Ig1•PTPσ Ig1-Ig2 complex on the IL1RAPL1•PTPδ Ig1-Ig2 complex, using the Ig1-Ig2 unit of the RPTP as the reference. Magnified view of the overlapped binding interfaces is also shown.

(c) Pair-wise superposition of the *trans*-synaptic type-IIa RPTP complexes, using the Ig1-Ig2 unit as the reference. Steric clashes are circled.

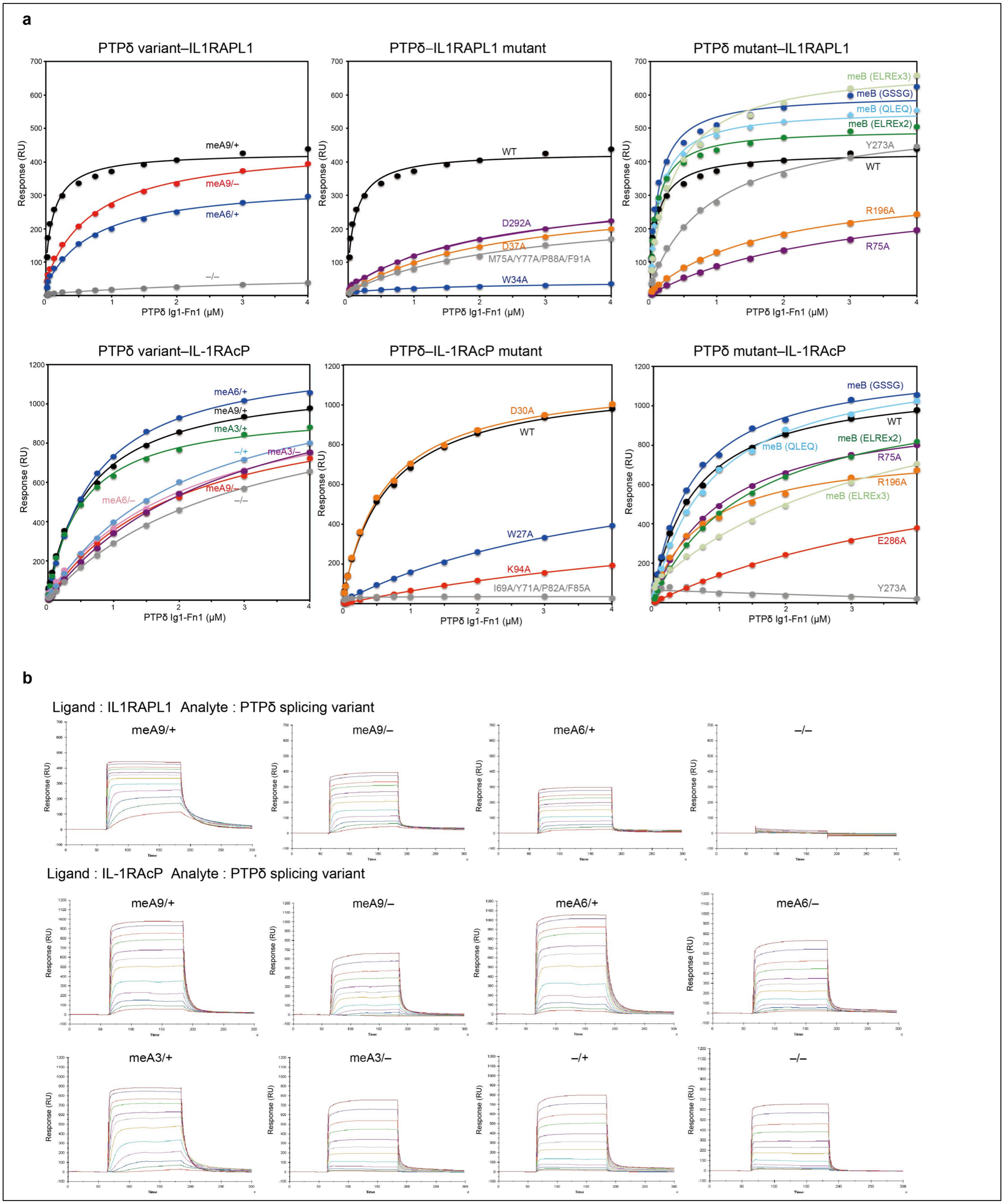
a
PTPδ variant–IL1RAPL1
meA9/+
meA9/–
meA6/+
–/–
PTPδ–IL1RAPL1 mutant
WT
D292A
D37A
M75A/Y77A/P88A/F91A
W34A
PTPδ mutant–IL1RAPL1
meB (ELREx3)
meB (GSSG)
meB (QLEQ)
meB (ELREx2)
Y273A
WT
R196A
R75A
Response (RU)
PTPδ Ig1-Fn1 (μM)
PTPδ variant–IL-1RAcP
meA6/+
meA9/+
meA3/+
–/+
meA3/–
meA6/–
meA9/–
–/–
PTPδ–IL-1RAcP mutant
D30A
WT
W27A
K94A
I69A/Y71A/P82A/F85A
PTPδ mutant–IL-1RAcP
meB (GSSG)
WT
meB (QLEQ)
meB (ELREx2)
R75A
R196A
meB (ELREx3)
E286A
Y273A
b
Ligand : IL1RAPL1 Analyte : PTPδ splicing variant
meA9/+
meA9/–
meA6/+
–/–
Ligand : IL-1RAcP Analyte : PTPδ splicing variant
meA9/+
meA9/–
meA6/+
meA6/–
meA3/+
meA3/–
–/+
–/–
Time

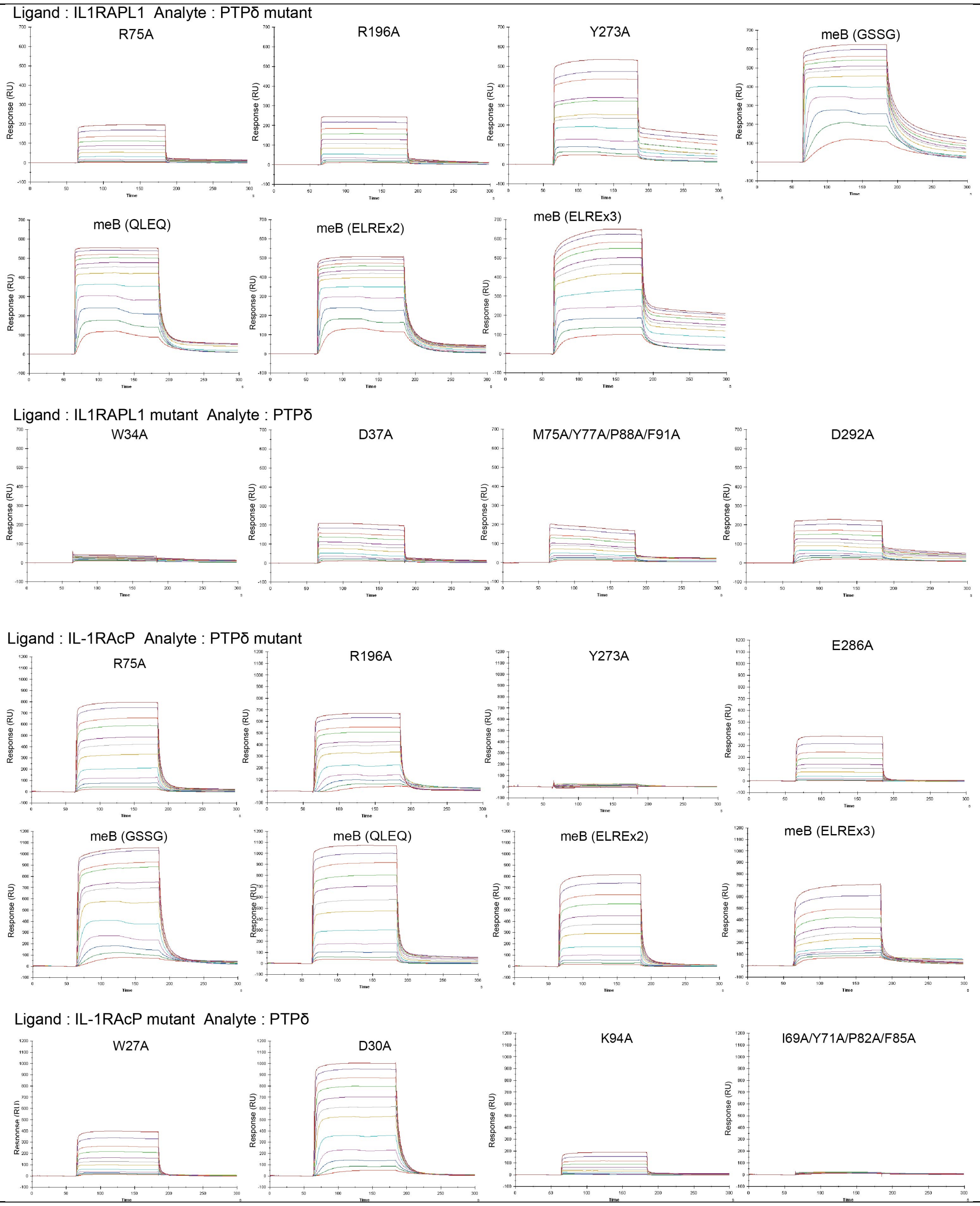

Supplementary Figure 4

SPR analysis of the interaction between PTPδ and IL1RAPL1/IL-1RAcP.

(a) SPR equilibrium measurements of the interaction between PTPδ Ig1-Fn1 and IL1RAPL1/IL-1RAcP. IL1RAPL1 or IL-1RAcP was immobilized on a CM5 sensor chip. Response units were plotted against concentrations of the indicative PTPδ Ig1-Fn1 proteins. The K_d values shown in Table 2-4 were calculated from these plots.

(b) SPR sensorgrams.
